# Platelets from patients with visceral obesity promote colon cancer growth

**DOI:** 10.1038/s42003-022-03486-7

**Published:** 2022-06-07

**Authors:** Marica Cariello, Elena Piccinin, Emanuela Pasculli, Maria Arconzo, Roberta Zerlotin, Simona D’Amore, Francesca Mastropasqua, Claudia Peres, Giusi Graziano, Gaetano Villani, Graziano Pesole, Antonio Moschetta

**Affiliations:** 1 Department of Interdisciplinary Medicine, “Aldo Moro” University of Bari, Bari, Italy; 2 INBB, National Institute for Biostructures and Biosystems, 00136 Rome, Italy; 3 Department of Basic Medical Sciences, Neurosciences and Sense Organs, “Aldo Moro” University of Bari, 70124 Bari, Italy; 4 Department of Medicine, Addenbrookes Hospital, University of Cambridge, Box 157, Hills Rd, Cambridge, CB2 0QQ UK; 5 Center of Neurodevelopmental Disorders (KIND), Division of Neuropsychiatry, Department of Women’s and Children’s Health, Karolinska Institutet, and Center for Psychiatry Research, Region Stockholm, Sweden; 6 Institute of Biomembranes, Bioenergetics and Molecular Biotechnologies, National Research Council (CNR), Bari, Italy

**Keywords:** Cancer models, Cancer microenvironment

## Abstract

Several studies highlighted the importance of platelets in the tumor microenvironment due to their ability to interact with other cell types such as leukocytes, endothelial, stromal and cancer cells. Platelets can influence tumor development and metastasis formation through several processes consisting of the secretion of growth factors and cytokines and/or via direct interaction with cancer cells and endothelium. Patients with visceral obesity (VO) are susceptible to pro-thrombotic and pro-inflammatory states and to development of cancer, especially colon cancer. These findings provide us with the impetus to analyze the role of platelets isolated from VO patients in tumor growth and progression with the aim to explore a possible link between platelet activation, obesity and colon cancer. Here, using xenograft colon cancer models, we prove that platelets from patients with visceral obesity are able to strongly promote colon cancer growth. Then, sequencing platelet miRNome, we identify *miR-19a* as the highest expressed miRNA in obese subjects and prove that *miR-19a* is induced in colon cancer. Last, administration of *miR-19a* per se in the xenograft colon cancer model is able to promote colon cancer growth. We thus elect platelets with their specific miRNA abundance as important factors in the tumor promoting microenvironment of patients with visceral obesity.

## Abstract (web summary)

Platelets from patients with visceral obesity, which highly express miR-19a, can strongly promote colon cancer growth in a xenograft colon cancer model, with miR-19a administration by AAV injection increasing tumor growth.

## Introduction

The tumor microenvironment is a chaotic and unregulated site where immune cells, fibroblasts, platelets, macrophages and endothelial cells deeply influence cancer cell metabolism. Platelets play a central role in thrombosis and hemostasis as well as in tissue repair and inflammation since they are able to directly interact with leukocytes, endothelial, stromal and intriguingly cancer cells^1–3^. Visceral obesity (VO) can be described as a metabolic condition that increases the risk of cardiovascular disease and cancer^4,5^. This condition, indeed, is characterized by inflammation and pro-thrombotic state that determine platelet hyperactivity^6^. Specifically, VO has been first linked to increased incidence of colorectal, prostate, pancreas, breast, kidney, esophagus and endometrial cancers^4^.

Platelets can influence tumor development and metastasis formation through several processes consisting of the secretion of growth factors and cytokines like TGF-β and PDGF^7^ and the direct interaction with cancer cells and endothelium. Specifically, colon cancer cell interaction with platelets promote metastasis dissemination in the lung inducing the production of TGF-β and the epithelial–mesenchymal like transition (EMT)^8^. At the same time, cancer cells are able to incorporate platelet-derived proteins promoting the dissemination of tumor into the bloodstream. In line with this, it has been postulated that the administration of low dose of aspirin decreases the incidence of cancer death probably reducing platelets/cancer cells cross-talk^9^. In this scenario, it is fundamental to understand the selective role of platelets in driving modulation of cancer metabolic events in the tumor microenvironment.

Here, using xenograft colon cancer models, we prove that platelets from patients with visceral obesity are able to strongly promote colon cancer growth. Then, sequencing platelet miRNome, we identify *miR-19a* as the highest expressed miRNA in obese subjects and prove that *miR-19a* is induced in colon cancer. Last, administration of *miR-19a* per se in the xenograft colon cancer model is able to promote colon cancer growth. We thus elect platelets with their specific miRNA abundance as important factors in tumor promoting microenvironment of patients with visceral obesity.

## Results and discussion

Visceral obesity (VO) can be described as a metabolic condition that increases the risk of cardiovascular disease and cancer^4,5^. In subjects with VO, platelets release inflammatory mediators such as PDGF, TGF-β and IL-1β that contribute to vascular inflammation and atherothrombosis^10^. Also, in patients with VO and type 2 diabetes, circulating platelet marker levels (P-selectin and CD40L) correlated with the enhancement of intima-media thickness (IMT)^11^. Thus, inflammation and atherothrombosis induced by platelets in VO have been postulated as a putative mechanism related to increased incidence of colon cancer in these subjects^4^. On a further relevance, patients with VO and metabolic disorders present an increased risk of mortality and recurrences for colon cancer^12^. However, the mechanisms by which obesity promotes tumor development and increased aggressivity are not fully elucidated, although several hypotheses have been proposed, including insulin resistance and inflammation^13^. In order to putatively disclose this conundrum, we decided to study the tumor-promoting ability of platelets of patients with VO. Twenty patients with VO were compared to twenty healthy subjects (clinical characteristics of the study population are in Supplementary Table 1). Compared to controls, VO patients were characterized by increased body weight, body mass index (BMI), waist circumference (WC) and blood pressure. As expected, VO patients displayed impaired glucose balance, dyslipidemia and increased markers of inflammation.

**Fig. 1:**
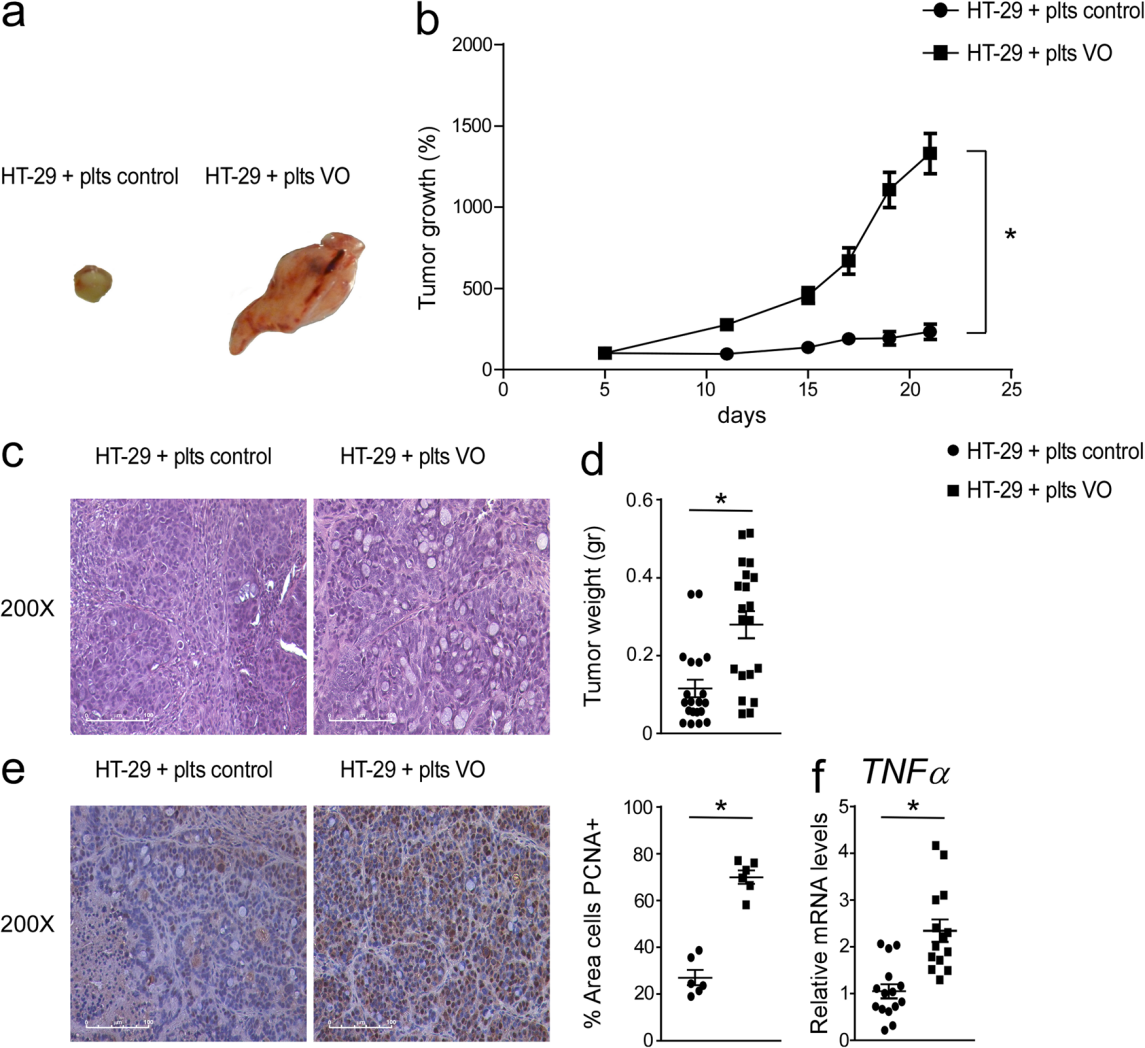
Platelets isolated from visceral obesity (VO) patients increased tumor growth in xenograft tumor model. **a** Athymic nu/nu mice were injected subcutaneously with HT29 cells and platelets isolated from VO patient and control were administered directly into the tumor mass every 7 days. Gross morphology of HT-29 cells treated with platelets isolated from VO patients and controls (*n* = 10 mice/group). The pictures were taken during the sacrifice with digital camera. **b** Tumor growth (%) curves showed high expansion of tumors injected with VO platelets (*n* = 20 tumors/group). **c** Histology was assessed by H&E staining and was observed by light microscopy (magnification, 200X). Representative specimens are shown. **d** Tumor weight (gr) was reported. **e** Paraffin-embedded tumor specimens from HT-29 cells inoculated with control platelets and HT-29 cells inoculated with VO platelets were immunoassayed with anti-PCNA antibody (200x magnification). Representative specimens are shown. PCNA staining per field was quantified by ImageJ software and displayed as percentage per field (*n* = 6 samples/group). **f** Gene expression analysis of *TNFα* in HT-29 cells inoculated with control platelets (*n* = 15) and HT-29 cells inoculated with VO platelets (*n* = 14). Cyclophilin was used as a housekeeping gene to normalize data. The results are shown as mean ± SEM in scatter plots. Statistical significance (*P* < 0.05) was assessed by student’s *t* test.

First, in xenograft tumor models, we observed a strongly significant increased growth of tumors inoculated with platelets isolated from VO patients compared to controls (Fig. 1a–d). To study the mechanisms involved in cancer cell proliferation, we examined the alterations of cell cycle and inflammation. Inoculation of VO platelets in colon cancer tumor xenografts significantly increased proliferating cell nuclear antigen (PCNA) protein accumulations and *TNFα* gene expression compared to treatment with control platelets (Fig. 1e, f), underscoring the central role of VO platelets in both inflammation and colon cancer cell proliferation. TNF-α is a key inflammatory cytokine present in the tumor microenvironment and it is involved in promotion and progression of several tumors. In colon cancer model, it has been demonstrated that TNF-α may promote tumor invasion via the ERK1/2 signaling pathway^14^. Thus, we now know that VO platelets, which are the only cells in the tumor microenvironment of the xenograft colon cancer models, are clearly able to stimulate tumor growth compared to control platelets, indicating that the platelet content is affected by the clinical condition of the patients.

**Fig. 2:**
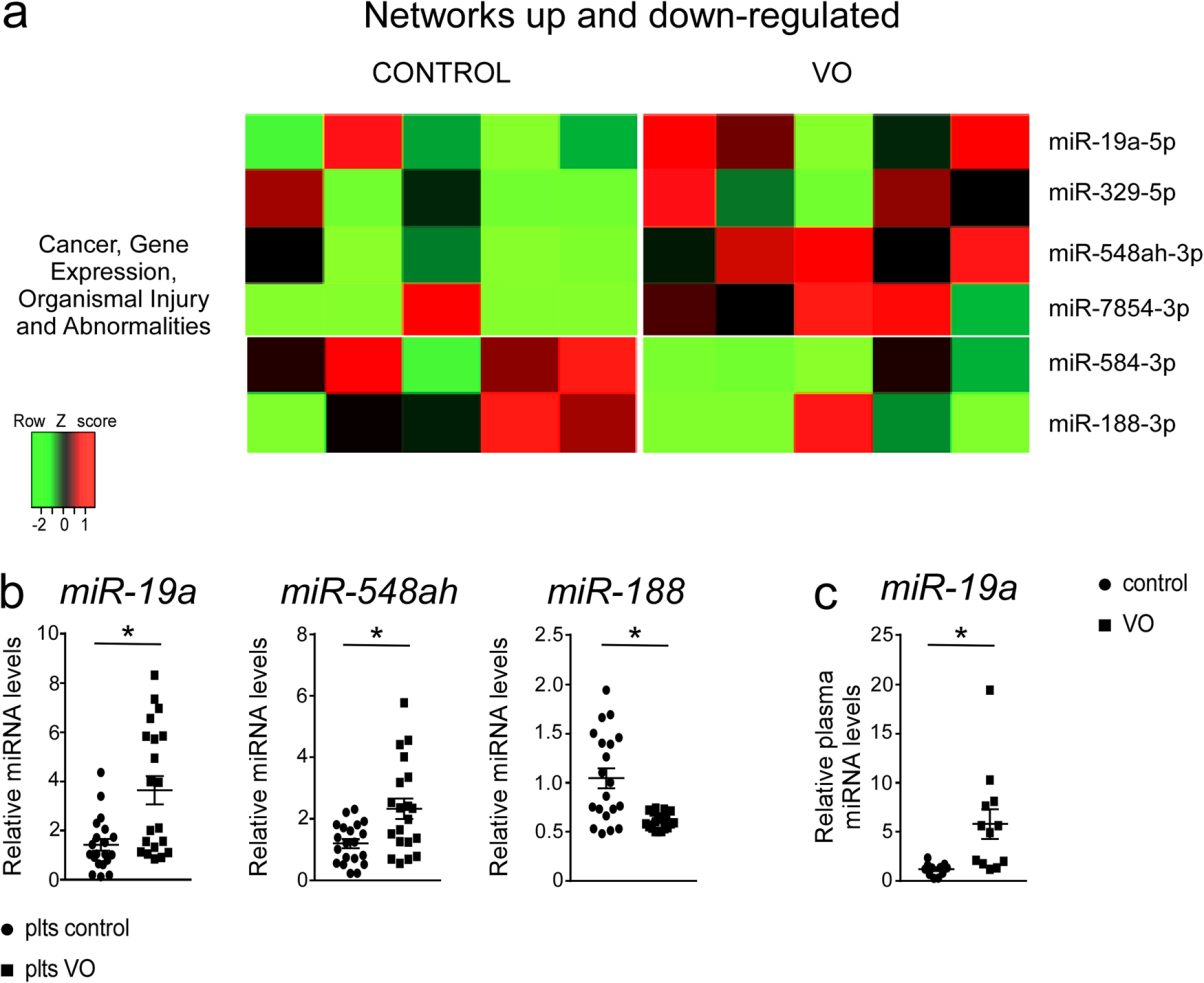
Identification of miRNome profile in platelets isolated from VO patients. **a** miRNAs up and down-regulated in platelets isolated from VO patients and controls were profiled using sequencing, and clustered in networks displaying a coordinate biological function. Data are shown in a heatmap with a matrix format of the miRNA differentially modulated within the network; single rows represent miRNA expression in a single patient (column). Colors: red, expression greater than the mean; black, expression equal to the mean; green, expression smaller than the mean (*n* = 5 samples/group). **b** Relative *miR-19a*, *miR-548ah* and *miR-188* expression from platelets of VO patients and controls (*n* = 20 samples/group). **c** Relative plasma *miR-19a* expression from VO patients and controls (*n* = 12 samples/group). The data were normalized on the geometric mean (best-keeper gene) of *miR-374* and *RNU6B*, presented as relative expression values. All results are shown as means ± SEM in scatter plots. Statistical significance (*P* < 0.05) was assessed by student’s *t* test.

Platelets are anucleated cells rich in protein and genetic material, including miRNAs that can be transferred to other cells^15^. MiRNAs are 20–22 nucleotides in length, single-strand RNAs that constitute a class of non-coding endogenous RNA and regulate gene expression inducing translational repression and/or mRNA degradation of their target gene^16^. miRNAs are implicated in several biological and pathological processes. Platelets receive miRNA processing protein and miRNA transcripts from their parental megakaryocytes^17^. It has been demonstrated that platelet miRNA profile is associated with platelet reactivity and platelets miRNAs are able to regulate more than 200 human genes^18^. We thus performed miRNA sequencing in platelets isolated from healthy controls and VO patients. We selected 50 annotated miRNAs that were differentially modulated in VO platelets compared to controls (*P* < 0.01; FDR < 0.05). To confer a framework for interpretation of our results, significant biological pathways were functionally clustered using the Core Function of Ingenuity System Pathway Analysis (IPA). We considered ‘biologically relevant’ only those ‘statistically significant’ miRNAs included in ‘significantly modulated’ networks (Fig. 2a). Compared to healthy subjects, in VO platelets there was a highly significant modulation of the miRNA category network involved in “Cancer, gene expression, organismal injury and abnormalities” (Fig. 2a). As shown in Fig. 2b, by RT-qPCR we confirmed the upregulation of *miR-19a-5p*, *miR-548ah-3p* and the down-regulation of *miR-188-3p* in platelets isolated from VO patients compared to those from healthy subjects. Recently, it has been demonstrated that *miR-548ah-3p* was significantly up-regulated in diabetic nephropathy patients^19^. Luo et al. observed that the upregulation of *miR-188-3p* reduced *N-myc downstream-regulated gene 1* (*NRDG1*) expression in hepatocellular carcinoma cell lines, which consequently inhibited cell growth and cell migration^20^. Specifically, we focused on *miR-19a-5p*, the most statistically significant and biologically relevant miRNA. Furthermore, in order to evaluate if *miR-19a* expression was systemically increased in these patients we measured plasma *miR-19a* expression observing a significant upregulation of *miR-19a-5p* in VO patients compared to healthy subjects (Fig. 2c). Our data clearly indicate that VO platelets displayed a peculiar miRNome pro-tumoral profile characterized by *miR-19a* overexpression as the most significant one. However, we can not exclude that other platelet factors from VO platelets that may synergistically contribute to the tumor growth. Indeed, due to technical difficulties with the xenograft model, our study lacks *miR-19a* silencing that could represent a powerful approach to determine the importance of this miRNA in VO platelets.

**Fig. 3:**
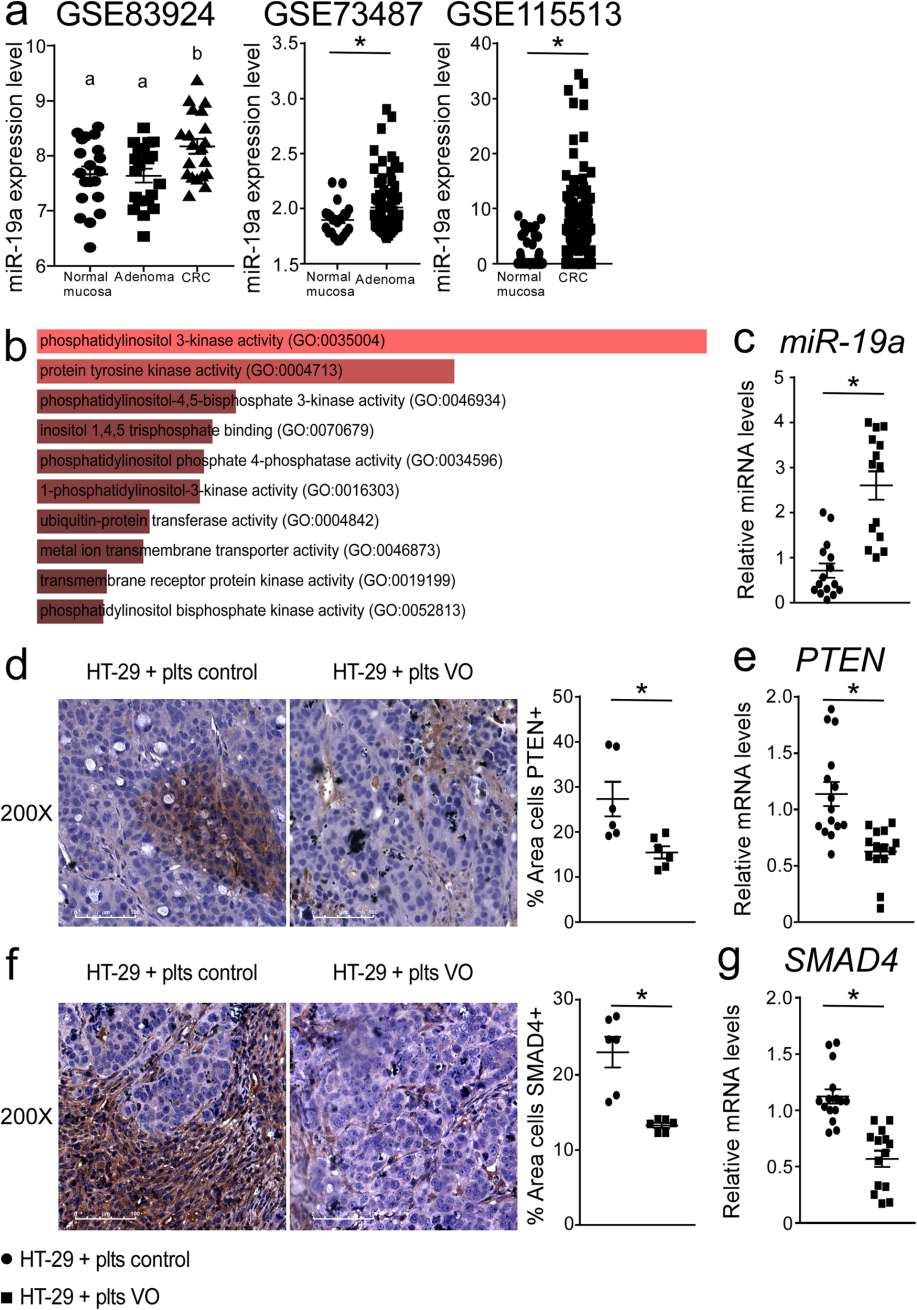
*miR-19a* upregulation in xenograft tumors treated with platelets isolated from VO patients. **a** *MiR-19a* expression level in normal mucosa, adenoma and CRC. Comparison of different groups was performed using One-way ANOVA followed by Tukey’s test for multiple comparison. The results are shown as mean ± SEM in scatter plots. Statistical significance of two groups was assessed by student’s *t* test. *P* value < 0.05 was considered significant. **b** MicroRNA target prediction and enrichment analysis of the predicted target genes. Genes targeted by *miR-19a* were predicted using TargetScan database. The gene set enrichment analysis was assessed using EnrichR. The bar graphs show the predicted mouse Kyoto Encyclopedia of Genes and Genomes (KEGG) pathways based on p– value ranking. **c** Relative *miR-19a* expression from HT-29 cells (xenograft tumor) treated with control platelets (*n* = 15) or VO platelets (*n* = 14). The data were normalized on the geometric mean (best-keeper gene) of *miR-374* and *RNU6B*, presented as relative expression values. Paraffin-embedded tumor specimens from HT-29 cells inoculated with control platelets and HT-29 cells inoculated with VO platelets were immunoassayed with (**d**) anti-PTEN and (**f**) anti-SMAD4 antibody (200x magnification). Representative specimens are shown. PTEN and SMAD4 staining per field was quantified by ImageJ software and displayed as percentage per field (*n* = 6 samples/group). The results are shown as mean ± SEM. Statistical significance (*P* < 0.05) was assessed by student’s *t* test. Gene expression analysis of (**e**) *PTEN* and (**g**) *SMAD4* in xenograft tumors treated with control platelets (*n* = 15) and xenograft tumors treated with VO platelets (*n* = 14). Cyclophilin was used as a housekeeping gene to normalize data. The results are shown as mean ± SEM in scatter plots. Statistical significance (*P* < 0.05) was assessed by student’s *t* test.

We thus decided to evaluate *miR-19a* expression in human colon cancer samples. We analyzed the *miR-19a* expression in colon cancer dataset using the gene expression omnibus (GEO) database. We collected three datasets (GSE83924, GSE73487, GSE115513) of miRNA sequencing in normal mucosa, adenoma and colon cancer (CRC). A significant increase in *miR-19a* expression levels were observed in CRC and adenoma samples compared to normal mucosa (Fig. 3a). Then, to understand the molecular mechanism by which *miR-19a* functions, we investigated *miR-19a* relevant KEGG pathways. We found that *miR-19a* was involved in 10 significant KEGG pathways (P < 0.05) including cancer related signaling pathways such as phosphatidylinositol 3-kinase activity, protein tyrosine kinase activity, ubiquitin-protein transferase activity (Fig. 3b).

*miR-19a* is the most important oncogenic member of the miR-17-92 cluster^21^. The overexpression of this cluster has been observed in several types of cancer like breast, lung, prostate, lymphoma and colon. In thyroid cancer, *miR19* upregulation reduced SMAD4 expression leading the cells less responsive to anti-proliferative effects of TGFβ^22^. Furthermore, overexpression of *miR-19* induced a strong reduction of the tumor suppressor PTEN levels, activating survival signaling (Akt/mTOR) and tumorigenesis^21,23^. In line with this, we observed a significant *miR-19a* upregulation with reciprocal down-regulation of its target genes *PTEN* and *SMAD4* in xenograft tumors inoculated with platelets isolated from VO patients compared to tumors with control platelets (Fig. 3c–g). Overall, these data clearly indicate that VO platelets displayed a peculiar miRNome profile that is able to promote intestinal tumor growth. One could speculate that modulation of platelet miRNome might represent a future strategy to reduce increased tumor development and aggressivity in obesity. Intriguingly, in healthy subjects and patients with VO, dietary nutrient modulation such as administration of high polyphenol extra virgin olive oil has been shown to decrease *miR-19a* expression levels in peripheral cells^24^.

**Fig. 4:**
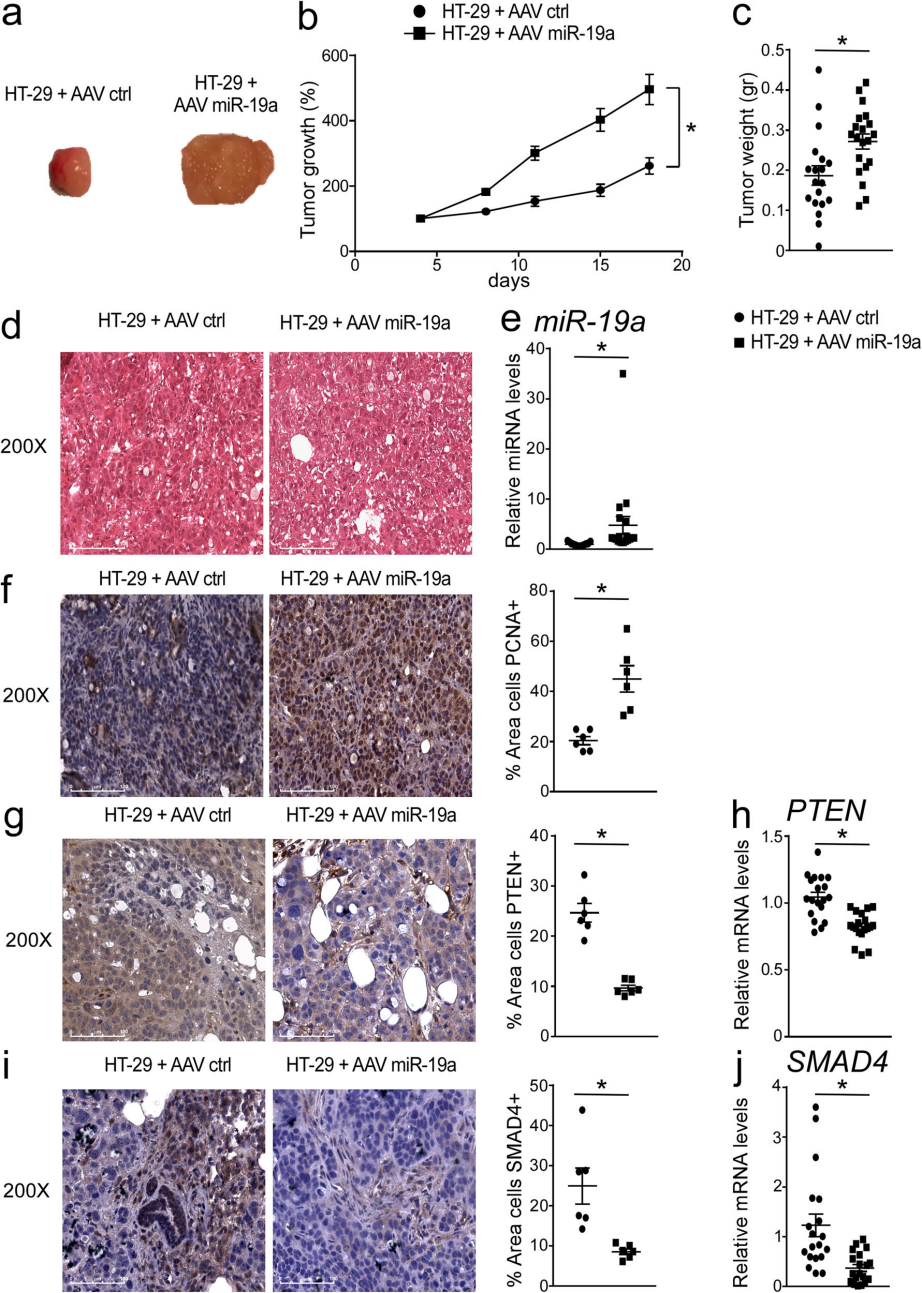
*miR-19a* increased tumor growth in xenograft tumor model. **a** Athymic nu/nu mice were injected subcutaneously with HT29 cells and a single injection of 5*10^10^ GC/ml of AAV-EGFP (control) or AAV-miR-19a suspension were administered directly into the tumor mass. Gross morphology of HT-29 cells treated with AAV-miR19a and control (*n* = 10 mice/group). The pictures were taken during the sacrifice with digital camera. **b** Tumor growth (%) curves showed high expansion of tumors injected with AAV-miR-19a (*n* = 20 tumors/group). **c** Tumor weight (gr) was reported (*n* = 19 HT-29 cells treated with AAV-ctrl vs *n* = 20 HT-29 cells treated with AAV-miR-19a). **d** Histology was assessed by H&E staining and was observed by light microscopy (magnification, ×200). Representative specimens are shown. **e** Relative *miR-19a* expression from xenograft tumors treated with AAV-EGFP and AAV-miR-19a (*n* = 19 samples/group). The data were normalized on the geometric mean (best-keeper gene) of *miR-374* and *RNU6B*, presented as relative expression values. Paraffin-embedded tumor specimens from HT-29 cells treated with AAV-EGFP and AAV-miR-19a were immunoassayed with (**f**) anti-PCNA, (**g**) anti-PTEN and (**i**) anti-SMAD4 antibody (×200 magnification). Representative specimens are shown. PCNA, PTEN and SMAD4 staining per field was quantified by ImageJ software and displayed as a percentage per field (*n* = 6 samples/group). Gene expression analysis of (**h**) *PTEN* and (**j**) *SMAD4* in xenograft tumors treated with AAV-EGFP (control) or AAV-miR-19a (*n* = 19 samples/group). Cyclophilin was used as a housekeeping gene to normalize data. All results are shown as means ± SEM in scatter plots. Statistical significance (*P* < 0.05) was assessed by student’s *t* test.

Finally, in order to evaluate the direct role of *miR-19a* in colon cancer development, we took advantage of a xenograft tumor model injected with miR-19a-5p AAV. The tumor growth curves and tumor weight revealed a significant increase in the expansion of tumors infected with AAV-miR-19a compared to tumors treated with AAV-EGFP (Fig. 4a–d). miR-19a AAV injection significantly increased PCNA protein accumulations (Fig. 4f). As expected, compared to controls in tumors treated with AAV-miR-19a we observed a significant increase of *miR-19a* expression levels (Fig. 4e) and a reciprocal down-regulation of its target genes *PTEN* and *SMAD4* (Fig. 4g–j). These data demonstrate that *miR-19a* is able to directly promote intestinal tumor growth, nevertheless, our study is limited to a single colon cancer HT-29 cell line and in the future more cancer cells should be used in xenograft model to validate our findings.

## Conclusions

We proved the pivotal role of platelets in the tumor microenvironment. Platelets transferred protein and genetic material to other cells and the platelet content is affected by the clinical condition of the subjects. Here we show that platelets isolated from VO patients are able to strongly induce intestinal tumor growth. Compared to platelets of healthy subjects, platelets from VO patients present peculiar miRNome profile. In particular, *miR-19a*, which is overexpressed in colon cancer and intestinal adenoma, was significantly induced in platelets of VO patients and in tumors inoculated with VO platelets. In xenograft tumor model, *miR-19a* per se was able to promote cell cycle progression and tumor development. Our study thus underscores the role of platelets in the intestinal tumor microenvironment driving colon cancer promoting scenario in visceral obesity. Moreover, the identification of tumor-promoting *miR-19a* as one of the relevant miRNA expressed in platelets elects platelet-specific miRNome as a putative novel bona fide strategy to prevent the development and/or to modify the prognosis of colon cancer.

## Methods

### Study population

Patient recruitment was performed in the Department of Interdisciplinary Medicine, Internal Medicine Division, “Aldo Moro” University of Bari, Italy. Twenty healthy subjects and 20 patients at the first diagnosis of Metabolic Syndrome and VO were recruited for this study (baseline characteristics are shown in Supplementary Table 1). The diagnosis of Metabolic Syndrome was assessed in accordance to the Adult Treatment Panel III (presence of three or more criteria). Waist Circumference (WC) was considered a marker of VO. Accordingly, VO is defined as WC > 102 cm for males or >88 cm for females. Cardiovascular risk was calculated using the scoring system of the Progetto Cuore. The study was conducted according to the Declaration of Helsinki Principles and the protocol was approved by the Ethics Committee of the Azienda Ospedaliero-Universitaria Policlinico di Bari, Italy. All patients gave their written informed consent.

### Xenograft Mouse Model

A colorectal cell suspension (100 µL; 1 × 10^7^ HT29 cells from the American Type Culture Collection, Manassas, VA) was injected subcutaneously into the subscapular region of an athymic mouse (CD1 nude mice; The Jackson Laboratory)^25^. Eight week-old female mice were used in each group of experiments. The tumors were not recovered until the diameter had increased to nearly 0.3–0.5 cm. The animals were treated with pool of platelets isolated from VO patients or controls and AAV-EGFP or AAV-miR-19a (Vector Biolabs, Malvern PA, USA). For xenograft treated with human platelets, mice were divided randomly into 2 groups, each group containing 10 mice (ie, 20 tumors) as follows: visceral obesity (VO) platelets group and healthy subject platelets group (control group). Subsequently, 100 µL of platelet suspension (10^6^platelets) was injected directly into the tumor-bearing mice in each group every 7 days for a total of 3 injections per mouse. For xenograft treated with AAV, mice were divided randomly into 2 groups (10 mice/group) as follows: AAV-miR-19a group and AAV-EGFP group (control group). A single injection of 5 × 10^10^ genome copies/mL (GC/ml) of AAV suspension was administered directly into the tumor-bearing mice in each group. Tumor growth was measured as long diameter (a) and short diameter (b) were calculated via a digital vernier caliper, and tumor volumes (V) were determined according to the formula *V* = ½·*a*·*b*^2^, and a tumor growth curve was drawn as percentage. All the mice, then were sacrificed and the tumors were harvested for further analysis. All mice were fed standard rodent chow diet and water *ad libitum* and kept under pathogen-free conditions in a temperature-controlled room (23 °C) on a 12-h light/dark cycle. The Ethical Committee of the University of Bari and Fondazione “Mario Negri” Sud authorized this protocol, which also was approved by the Italian Ministry of Health in accordance with internationally accepted guidelines for animal care.

### Platelets isolation

Platelets were isolated from whole blood and anti-coagulated with sodium citrate 3.8%. Platelet-rich plasma (PRP) was prepared by centrifugation of citrated blood at 200 g for 20 min at 25 °C. Platelets were sedimented at 1000 g for 15 min and then were washed with prostaglandin E1 (PGE1) (Sigma, Italy) and resuspended in ice-cold HEPES-Tyrode buffer (pH 7.4) containing 129 mM NaCl, 8.9 mM NaHCO3, 2.8 mM KCl, 0.8 mM KH2PO4, 56 mM dextrose, 10 mM HEPES. After sedimentation, the platelets were washed twice in this buffer. To isolate the platelets it has been used HEPES as the pH buffer in Tyrode’s solution. pH buffers such as Tris (tris[hydroxymethyl]aminomethane) should be avoided because, like other amines, they inhibit some platelet responses and potentiate others.

### MiRNA Sequencing

MicroRNA-Seq libraries were prepared from 100 ng of small RNA isolated from platelets samples, according to the Illumina TruSeq Small RNA Sample Preparation Protocol (Illumina). MiRNA-Seq libraries were pooled in equimolar ratios (4 libraries per run), denatured and diluted to a final concentration of 10 pM and sequenced on the Illumina MiSeq platform, generating approximately 2 to 4 million 50 bp reads/sample. Adapter removal was performed using a custom Perl script. Expression levels were assessed by aligning reads between 17 and 25 bp in size to the complete collection of mature human miRNAs (obtained from miRBase, version 21) allowing up to 1 mismatch. Read counts were normalized using the trimmed mean normalization, as applied in the edgeR package. Differential expression analysis was carried out using the latest version of the limma (v3.32) and the edgeR (v3.18) packages in R. Prediction of miRNA targets was performed by using TargetScan. The gene set enrichment analysis was performed using the EnrichR package. Sequencing data are freely available through the SRA BioProject PRJNA820035.

### RNA and miRNA Extraction

Total RNA was extracted by Qiazol reagent (Qiagen) following the manufacturer’s instructions. cDNA was synthesized retrotranscribing 4 μg of total RNA in a total volume of 100 μL using a High Capacity DNA Archive Kit (Thermo Fisher Scientific) for the manufacturer’s instructions. MiRNA isolation was performed using mirVana™ miRNA Isolation Kit (Thermo Fischer, MA, USA) following the manufacturer’s guidelines. Plasma miRNA isolation was performed using miRNaesy serum/plasma advanced kit (Qiagen, GmbH) following the manufacturer’s guidelines. RNA quality was assessed by 260 nm absorbance with Nanodrop spectrophotometer (NanoDrop Technologies Inc., Wilmington, DE, USA) and by capillary electrophoresis with an Agilent2100 Bioanalyzer (Agilent Technologies, Santa Clara, CA, USA). For miRNA expression analysis, reverse transcription was made using TaqMan microRNA Reverse Transcription Kit (Thermo Fisher Scientific, MA, USA), following the manufacturer’s instructions.

### Real-time quantitative PCR

For genes: Real-time quantitative PCR (RTqPCR) primers were designed using Primer Express software. Cyclophilin was used as internal control. Validated primer sequences for RTqPCR are: *TNFα* FW CCTCTGGCCCAGGCAGT RV AGGCTTGTCACTCGGGGTT; *cyclophilin* FW CATCTGCACTGCCAAGACTGAG RV CCTTCACTTTGCCAAACACCAC. For *PTEN* and *SMAD4* RTqPCR we used an IDT validated primer (*PTEN* code: Hs.PT.53.26587300; *SMAD4* code: Hs.PT.58.472774). For miRNAs: qPCR assays were made using pre-validated TaqMan Assays and TaqMan Universal Master Mix (Thermo Fisher Scientific, MA, USA), following the manufacturer instructions. Quantitative normalization was performed using *miR-374* and *RNU6B* as internal controls. For all experiments the PCR assays were performed in 96well optical reaction plates using the QuantStudio5 machine (Thermo Fisher Scientific). Relative quantification was performed via the ΔΔCT method.

### Histology and immunohistochemistry

Tissue specimens were fixed in 10% formalin for 12–24 h, dehydrated, and paraffin embedded. 4 µm thick sections were stained with hematoxylin-eosin (H&E) following standard protocol. For antigen retrieval slides were boiled in sodium citrate pH 6 (Sigma Aldrich, Milan, Italy) for 15 min. Sections were permeabilized in phosphate-buffered saline (PBS) with 0.25% TritonX-100 for 5 min and then were incubated at room temperature in protein blocking solution for 10 min (Dako, Glostrup, Denmark) and overnight at 4 °C with the primary antibodies (anti-pcna 1:100, Santa Cruz Biotechnology, Santa Cruz, CA; or anti-PTEN 1:2000, Abcam, Cambridge, UK or anti-SMAD4 1:400, Abcam, Cambridge, UK). Sections were rinsed in PBS (15 min) and incubated at room temperature with DAKO real EnVision detection system Peroxidase/DAB^+^ (25 min, Dako, Glostrup, Denmark) according to manufacturer’s instruction. Image analysis was made through Image J software. For each sample, 10 representative images were acquired with a 20x objective. The percentage of stained area/total area was evaluated. Values from all consecutive images for each sample were averaged. For negative controls, 1% nonimmune serum in PBS substituted the primary antibodies.

### Gene expression Omnibus analysis

We evaluated potential GEO datasets according to the following inclusion parameters: (1) histological diagnosis; (2) colorectal cancer or adenoma for the experimental group; (3) normal intestinal mucosa used as controls; (4) Non-coding RNA profiling by array and raw data required the CEL or TXT format; (5) performed on the GPL18402 (Agilent-046064Unrestricted_Human_miRNA_V19.0_Microarray) and Affymetrix Multispecies miRNA-3 Array. Datasets with specimens from other organisms, expression profiling by RT-PCR, or sample size <60 were excluded. We used the search terms colorectal cancer OR crc AND homo sapiens AND microrna microarray in the GEO DataSets to identify potential datasets. Then, we further screened these datasets according to the above inclusion criteria. Finally, 3 GEO datasets, GSE83924, GSE73487, and GSE115513, were included in our study.

### Statistics and reproducibility

All results are expressed as mean ± SEM in scatter plots. Data distribution and gene expression statistical analysis were analyzed using GraphPad Prism software (v5.0; GraphPad Software Inc., San Diego, CA). Comparisons of two groups were performed using a Student’s *t* test. Comparison of different groups was performed using One-way ANOVA followed by Tukey’s test for multiple comparison. A *p* value of <0.05 was considered significant.

### Reporting summary

Further information on research design is available in the Nature Research Reporting Summary linked to this article.

## Supplementary information

Supplementary Information

Description of Additional Supplementary Files

Supplementary Data 1

Supplementary Data 2

Supplementary Data 3

Supplementary Data 4

Reporting Summary

## Data Availability

Sequencing data are freely available through the SRA BioProject PRJNA820035. Source data are provided as a supplementary data file (Supplementary Data 1–4). All other data are available from the corresponding author on reasonable request.
