# Supplementary material for: Platelets from patients with visceral obesity promote colon cancer growth
Source: Commun Biol. 2022 Jun 7;5:553. doi: 10.1038/s42003-022-03486-7 (PMC9174292; doi:10.1038/s42003-022-03486-7)
Supplement: Supplementary file 1 — Supplementary Information [file 42003_2022_3486_MOESM1_ESM.pdf]

Supplementary Table 1. Clinical characterization of the study population

Clinical variable	Control	VO	p-value
n (M:F)	20 (9:11)	20 (9:11)	-
Weight (Kg)	67.0±3.6	87.3±3.6	<0.01
WC (cm)	85.8±2.0	105.2±2.4	<0.01
BMI (Kg/m^2)	23.0±0.6	30.4±1.2	<0.01
SBP (mm Hg)	110.6±2.8	127.1±2.4	<0.01
DBP (mm Hg)	68.9±1.5	86.7±1.9	<0.01
Total cholesterol (mg/dl)	183.3±7.1	188.8±7.1	NS
HDL-C (mg/dl)	65.3±4.2	47.3±2.5	<0.01
LDL-C (mg/dl)	111.0±6.4	124.5±6.9	NS
TG (mg/dl)	63.4±6.4	119.4±10	<0.01
Glucose (mg/dl)	82.6±1.2	101.2±2.3	<0.01
HbA1c (%)	5.2±0.1	6.1±0.1	<0.01
Insulin (microUI/ml)	6.7±0.7	14.6±2.4	<0.01
HOMA-IR	1.3±0.2	3.8±0.6	<0.01
AST (U/I)	20.8±2	22.7±2	NS
ALT (U/I)	34.5±2.4	49.8±5.4	<0.05
CRP (mg/dl)	0.2±0	0.8±0.1	<0.01
Fibrinogen (mg/dl)	206.9±11.1	266.1±14	<0.01
Creatinine (mg/dl)	1.2±0.4	0.8±0	NS
Sodium (mEq/I)	139.9±0.6	135.8±4.7	NS
Potassium (mEq/I)	4.0±0.1	4.2±0.1	NS
Magnesium (mEq/I)	1.9±0.1	2.1±0.1	NS
Calcium (mg/dl)	9.0±0.1	8.8±0.2	NS
Phosphorus (mg/dl)	3.7±0.1	3.5±0.1	NS
WBC (10^3/µl)	6.4±0.4	6.9±0.4	NS
Platelets (10^3/µl)	253.5±11.3	280.2±11.5	NS
Monocytes (%)	6.4±0.4	5.6±0.3	NS
Lymphocytes (%)	37.1±1.2	33.3±1.4	NS
Neutrophils (%)	52.1±2.1	57.4±3.3	NS
Eosinophils (%)	2.9±0.4	2.4±0.2	NS
Basophils (%)	0.5±0.1	0.5±0	NS

Data are presented as mean ± SEM. Abbreviations: Waist Circumference, WC; Body Mass Index, BMI; systolic blood pressure, SBP; diastolic blood pressure, DBP; high-density lipoprotein cholesterol, HDL-C; low-density lipoprotein cholesterol, LDL-C; triglyceride, TG; homeostatic model assessment for insulin resistance, HOMA-IR; aspartate transaminase, AST; alanine transaminase, ALT; C-reactive protein, CRP; White blood cell, WBC; non-significant, NS.
