# Supplementary material for: Platelets from patients with visceral obesity promote colon cancer growth
Source: Commun Biol. 2022 Jun 7;5:553. doi: 10.1038/s42003-022-03486-7 (PMC9174292; doi:10.1038/s42003-022-03486-7)
Supplement: Supplementary file 2 — Description of Additional Supplementary Files [file 42003_2022_3486_MOESM2_ESM.pdf]

Description of Additional Supplementary Files

File name: Supplementary Data 1
Description: Source data for: tumor weight (Figure 1D), PCNA immunohistochemistry (Figure 1E), gene expression analysis of TNFα (Figure 1F).

File name: Supplementary Data 2
Description: Source data for: miR-19a, miR-548ah and miR-188 expression levels (Figure 2B), plasma miR-19a

File name: Supplementary Data 3
Description: Source data for: miR-19a expression levels (Figure 3C), PTEN immunohistochemistry (Figure 3D), gene expression analysis of PTEN (Figure 3E), SMAD4 immunohistochemistry (Figure 3F), gene expression analysis of SMAD4 (Figure 3G).

File name: Supplementary Data 4
Description: Source data for: tumor weight (Figure 4C), miR-19a expression levels (Figure 4E), PCNA immunohistochemistry (Figure 4F), PTEN immunohistochemistry (Figure 4G), gene expression analysis of PTEN (Figure 4H), SMAD4 immunohistochemistry (Figure 4I), gene expression analysis of SMAD4 (Figure 4J).
