# Supplementary material for: Platelets from patients with visceral obesity promote colon cancer growth
Source: Commun Biol. 2022 Jun 7;5:553. doi: 10.1038/s42003-022-03486-7 (PMC9174292; doi:10.1038/s42003-022-03486-7)
Supplement: Supplementary file 7 — Reporting Summary [file 42003_2022_3486_MOESM7_ESM.pdf]

nature portfolio

Corresponding author(s): Antonio Moschetta

Last updated by author(s): Mar 25, 2022

Reporting Summary

Nature Portfolio wishes to improve the reproducibility of the work that we publish. This form provides structure for consistency and transparency in reporting. For further information on Nature Portfolio policies, see our Editorial Policies and the Editorial Policy Checklist.

Statistics

For all statistical analyses, confirm that the following items are present in the figure legend, table legend, main text, or Methods section.

n/a	Confirmed	
☐	☒	The exact sample size (*n*) for each experimental group/condition, given as a discrete number and unit of measurement
☒	☐	A statement on whether measurements were taken from distinct samples or whether the same sample was measured repeatedly
☐	☒	The statistical test(s) used AND whether they are one- or two-sided<br>*Only common tests should be described solely by name; describe more complex techniques in the Methods section.*
☒	☐	A description of all covariates tested
☐	☒	A description of any assumptions or corrections, such as tests of normality and adjustment for multiple comparisons
☐	☒	A full description of the statistical parameters including central tendency (e.g. means) or other basic estimates (e.g. regression coefficient) AND variation (e.g. standard deviation) or associated estimates of uncertainty (e.g. confidence intervals)
☒	☐	For null hypothesis testing, the test statistic (e.g. *F*, *t*, *r*) with confidence intervals, effect sizes, degrees of freedom and *P* value noted<br>*Give P values as exact values whenever suitable.*
☒	☐	For Bayesian analysis, information on the choice of priors and Markov chain Monte Carlo settings
☒	☐	For hierarchical and complex designs, identification of the appropriate level for tests and full reporting of outcomes
☒	☐	Estimates of effect sizes (e.g. Cohen's *d*, Pearson's *r*), indicating how they were calculated

Our web collection on statistics for biologists contains articles on many of the points above.

Software and code

Policy information about availability of computer code

Data collection	Data collection was performed using GraphPad Prism software (v5.0; GraphPad Software Inc., San Diego, CA).
Data analysis	Data distribution and gene expression statistical analysis were analyzed using GraphPad Prism software (v5.0; GraphPad Software Inc., San Diego, CA).

For manuscripts utilizing custom algorithms or software that are central to the research but not yet described in published literature, software must be made available to editors and reviewers. We strongly encourage code deposition in a community repository (e.g. GitHub). See the Nature Portfolio guidelines for submitting code & software for further information.

Data

Policy information about availability of data

All manuscripts must include a data availability statement. This statement should provide the following information, where applicable:

- Accession codes, unique identifiers, or web links for publicly available datasets
- A description of any restrictions on data availability
- For clinical datasets or third party data, please ensure that the statement adheres to our policy

Sequencing data are freely available through the SRA BioProject PRJNA820035.

Field-specific reporting

Please select the one below that is the best fit for your research. If you are not sure, read the appropriate sections before making your selection.

☒ Life sciences ☐ Behavioural & social sciences ☐ Ecological, evolutionary & environmental sciences

For a reference copy of the document with all sections, see nature.com/documents/nr-reporting-summary-flat.pdf

Life sciences study design

All studies must disclose on these points even when the disclosure is negative.

Sample size	In order to calculate the sample size of the study we assumed to detect a difference of at least 50% in the tumor growth values between HT-29 cells treated with platelets isolated from VO patients and controls, a significance level of 0.05 and power of 90% obtaining a total of 10 mice/group.
Data exclusions	Exclusion criteria: renal and hepatic failure, acute heart diseases (cardiac failure, coronary arterial disease, acute arrhythmias), infections, neoplastic diseases with recent onset and/or under chemotherapy.
Replication	N/A
Randomization	Mice were divided randomly into groups.
Blinding	Investigators were blinded to group allocation during treatment and analysis.

Reporting for specific materials, systems and methods

We require information from authors about some types of materials, experimental systems and methods used in many studies. Here, indicate whether each material, system or method listed is relevant to your study. If you are not sure if a list item applies to your research, read the appropriate section before selecting a response.

Materials & experimental systems

n/a	Involved in the study	
☐	☒	Antibodies
☐	☒	Eukaryotic cell lines
☒	☐	Palaeontology and archaeology
☐	☒	Animals and other organisms
☐	☒	Human research participants
☒	☐	Clinical data
☒	☐	Dual use research of concern

Methods

n/a	Involved in the study	
☒	☐	ChIP-seq
☒	☐	Flow cytometry
☒	☐	MRI-based neuroimaging

Antibodies

Antibodies used

anti-pcna, sc-7907, Santa Cruz Biotechnology, Santa Cruz, CA; anti-pten, ab267787 Abcam, Cambridge, UK; anti-smad4 ab40759 Abcam, Cambridge, UK.

Validation

PCNA sc-7907 is recommended for detection of PCNA of mouse, rat, human, insect, Drosophila, Xenopus and zebrafish origin by immunohistochemistry (including paraffin-embedded sections) (starting dilution 1:50, dilution range 1:50-1:500).

References

1. Sasaki, T., et al. 2000. Colorectal carcinomas in mice lacking the catalytic subunit of PI(3)Kγ. Nature 406: 897-902.
2. Scotti, L., et al. 2011. Administration of a gonadotropin-releasing hormone agonist affects corpus luteum vascular stability and development and induces luteal apoptosis in a rat model of ovarian hyperstimulation syndrome. Mol. Cell. Endocrinol. 335: 116-125.
3. Johnson, M., et al. 2011. IQGAP1 translocates to the nucleus in early S-phase and contributes to cell cycle progression after DNA replication arrest. Int. J. Biochem. Cell Biol. 43: 65-73.
4. Belibi, F., et al. 2011. mTORC1/2 and rapamycin in female Han:SPRD rats with polycystic kidney disease. Am. J. Physiol. Renal Physiol. 300: F236-F244.
5. Lee, J.Y., et al. 2011. Curcumin induces EGFR degradation in lung adenocarcinoma and modulates p38 activation in intestine: the versatile adjuvant for gefitinib therapy. PLoS ONE 6: e23756.
6. Fang, Y., et al. 2012. TGF-β promotes proliferation of thyroid epithelial cells in IFN-γ-/- mice by down-regulation of p21 and p27 via AKT pathway. Am. J. Pathol. 180: 650-660.
7. Lopitz-Otsoa, F., et al. 2012. Integrative analysis of the ubiquitin proteome isolated using tandem ubiquitin binding entities (TUBEs). J. Proteomics 75: 2998-3014.
8. Yang, W., et al. 2012. ERK1/2-dependent phosphorylation and nuclear translocation of PKM2 promotes the Warburg effect. Nat. Cell Biol. 14: 1295-1304.
9. Sauvat, F., et al. 2013. Ovarian function is restored after grafting of cryopreserved immature ovary in ewes. FASEB J. 27:

1511-1518.
datasheet link: https://www.scbt.com/it/p/pcna-antibody-fl-261

anti-pten ab267787 is suitable for WB, IHC-P, IP, Flow Cyt (Intra). Positive control IHC: Human endometrial cancer, Human ovarian cancer, Human pancreas, Mouse pancreas and Rat pancreas tissues.
References
Wu Z et al. The inhibitory effect of human DEFA5 in growth of gastric cancer by targeting BMI1. Cancer Sci 112:1075-1083 (2021)
Zhou X et al. Silencing of MEG3 attenuated the role of lipopolysaccharides by modulating the miR-93-5p/PTEN pathway in Leydig cells. Reprod Biol Endocrinol 19:33 (2021).
Cheng Z et al. Upregulation of circRNA_100395 sponges miR-142-3p to inhibit gastric cancer progression by targeting the PI3K/AKT axis. Oncol Lett 21:419 (2021).
Zhan D et al. Hypoxia-inducible factor-1a regulates PI3K/AKT signaling through microRNA-32-5p/PTEN and affects nucleus pulposus cell proliferation and apoptosis. Exp Ther Med 21:646 (2021).
Yang H et al. LncRNA JPX regulates proliferation and apoptosis of nucleus pulposus cells by targeting the miR-18a-5p/HIF-1a/Hippo-YAP pathway. Biochem Biophys Res Commun 566:16-23 (2021).
datasheet link: https://www.abcam.com/pten-antibody-epr22636-122-ab267787.html

anti-smad4 ab40759 is suitable for WB, IHC-P. Positive control IHC-P: Human lung carcinoma and breast carcinoma tissues.
References
Long C et al. FOXO3 is targeted by miR-223-3p and promotes osteogenic differentiation of bone marrow mesenchymal stem cells by enhancing autophagy. Hum Cell 34:14-27 (2021).
Zhao S et al. Effects of Smad4 on the expression of caspase-3 and Bcl-2 in human gingival fibroblasts cultured on 3D PLGA scaffolds induced by compressive force. Int J Mol Med 47:N/A (2021).
Zhang T et al. Proteomics reveals the function reverse of MPSSS-treated prostate cancer-associated fibroblasts to suppress PC-3 cell viability via the FoxO pathway. Cancer Med 10:2509-2522 (2021).
Wang W et al. Positive feedback regulation between USP15 and ERK2 inhibits osteoarthritis progression through TGF-ß/SMAD2 signaling. Arthritis Res Ther 23:84 (2021)
Xu R et al. MicroRNA-181a promotes epithelial-mesenchymal transition in esophageal squamous cell carcinoma via the TGF-ß/Smad pathway. Mol Med Rep 23:N/A (2021).
datasheet link: https://www.abcam.com/smad4-antibody-ep618y-ab40759.html

Eukaryotic cell lines

Policy information about cell lines

Cell line source(s)	HT29 cells were obteined from the American Type Culture Collection, Manassas, VA.
Authentication	We declare that we have not authenticated the cells purchased from ATCC
Mycoplasma contamination	I confirm that HT29 line tested negative for mycoplasma contamination.
Commonly misidentified lines (See ICLAC register)	N/A

Animals and other organisms

Policy information about studies involving animals; ARRIVE guidelines recommended for reporting animal research

Laboratory animals	We used athymic male mouse (CD1 nude mice; The Jackson Laboratory) 8 weeks old.
Wild animals	N/A
Field-collected samples	All mice were fed standard rodent chow diet and water ad libitum and housed under pathogen-free conditions in a temperature-controlled room (23°C) on a 12-hour light/dark cycle. At the end of the experiments, mice were sacrificed and the tumors were harvested for further analysis.
Ethics oversight	The Ethical Committee of the University of Bari and Fondazione "Mario Negri" Sud approved this protocol, which also was authorized by the Italian Ministry of Health in accordance with internationally accepted guidelines for animal care.

Note that full information on the approval of the study protocol must also be provided in the manuscript.

Human research participants

Policy information about studies involving human research participants

Population characteristics	Baseline characteristics are shown in Supplementary Table 1.
Recruitment	Patient recruitment was performed in the Department of Interdisciplinary Medicine, Internal Medicine Division, "Aldo Moro" University of Bari, Italy. Twenty healthy subjects and twenty patients at the first diagnosis of Metabolic Syndrome and visceral obesity (VO) were recruited for this study. The diagnosis of Metabolic Syndrome was assessed in accordance to the Adult Treatment Panel III (presence of three or more criteria). Waist Circumference (WC) was considered a marker of VO. Accordingly, VO is defined as WC >102 cm for males or >88 cm for females.

Ethics oversight	The study was conducted according to the Declaration of Helsinki Principles and the protocol was approved by the Ethics Committee of the Azienda Ospedaliero-Universitaria Policlinico di Bari, Italy.

Note that full information on the approval of the study protocol must also be provided in the manuscript.
